# The use of electronic nicotine delivery systems during pregnancy and the reproductive outcomes: A systematic review of the literature

**DOI:** 10.18332/tid/104724

**Published:** 2019-07-01

**Authors:** Victor M. Cardenas, Lori A. Fischbach, Parimal Chowdhury

**Affiliations:** 1 University of Arkansas for Medical Sciences, Little Rock, United States

**Keywords:** pregnancy, risk, prevalence, electronic nicotine delivery systems (ENDS), reproductive outcomes

## Abstract

**INTRODUCTION:**

Use of electronic nicotine delivery systems (ENDS) among pregnant women is of great concern. To our knowledge the current literature provides conflicting views regarding the uncertainties of the effects of ENDS use during pregnancy on the health of the fetus.

**METHODS:**

We searched PubMed, CINAHL, and EMBASE, for the period 2007 to October 2017 for terms to identify publications on ENDS use during pregnancy and the reproductive outcomes. We updated the search for the period November 2017 to November 2018 using Ovid Medline. We obtained full text of articles and present a summary of the contents.

**RESULTS:**

We found no studies of pregnant women exposed to ENDS use and its effect on their fetus or neonates. However, there is a growing body of experimental studies in animals that suggest that nicotine in ENDS alters DNA methylation, induces birth defects, reduces the birth weight, and affects the development of the heart and lungs of their offspring. A large population-based cohort study in the United States estimated that 5% of pregnant women were current ENDS users in 2014; most of them also smoked cigarettes. Surveys conducted among practitioners indicate that there is a need to screen and counsel pregnant women. Systematic reviews and meta-analysis of studies of women who used smokeless tobacco during pregnancy suggest that prenatal nicotine alone is a risk factor for low birth weight, premature delivery, and stillbirth.

**CONCLUSIONS:**

There were no previous studies assessing the reproductive effects of ENDS use during pregnancy. However, prenatal exposure to nicotine is known to be harmful to the fetus and the pregnancy.

## INTRODUCTION

According to the USA Food and Drug Administration, electronic nicotine delivery systems (ENDS) are defined as electronic devices that deliver e-liquid in aerosol form into the mouth and lungs when inhaled. ENDS were introduced to the US market in 2006, and by 2014 had overtaken cigarette smoking as the lead tobacco product used by teenagers, and although teen use declined in 2016, it resurged during 2017–18 with the widespread use of the USB-like device that uses a rechargeable cartridge or pod, hence their description as mod-pods, or by its leading brand name, JUUL^1^. Therefore, it is possible that in the future a significant proportion of young pregnant women could be exposed to this emerging tobacco product. ENDS have been marketed as healthier alternatives to combustible tobacco^2^ because ENDS e-liquids and aerosols have been found to contain fewer toxicants and carcinogens than those found in tobacco smoke. In particular, no carbon monoxide and other combustion by-products have been found in ENDS e-liquids and aerosols^3^.

A study^4^ illustrated that nicotine crosses the placenta by showing a strong correlation between the levels of oral cotinine in pregnant women and the presence of nicotine and its metabolites in foetal compartments and meconium. That study further showed a strong association between the presence of nicotine and nicotine biomarkers in meconium and adverse reproductive outcomes^4^. Prenatal nicotine exposure in animal models has been shown to affect the vascularization of the placenta, resulting in decreased decidua and junctional zone area. This exposure also appears to decrease the expression and production of angiogenic factors, which results in limited differentiation of trophoblasts, and the expression of placental nicotinic acetylcholine receptors^5^.

Although most professional organizations such as the American College of Obstetricians and Gynecologists discourage pregnant women from exposure to nicotine, and express caution about the use of nicotine replacement therapy, due to its known deleterious effects on the fetus^6^, the USA National Academy of Sciences concluded in its 2018 report that ‘little can be said…regarding the potential effects of e-cigarettes…on pregnancy outcomes’. Moreover, a report^7^ of the US National Academy of Sciences reached two conclusions on the topic: 1) ‘There is no available evidence whether or not e-cigarettes affect pregnancy outcomes’, and 2) ‘There is insufficient evidence whether or not maternal e-cigarette use affects fetal development’. The uncertainty of the effects of ENDS use during pregnancy could lead to inaction, hence the authors felt compelled to summarize studies regarding ENDS use during pregnancy to identify and provide a summary of what is known about the effects of other non-combustible tobacco products on reproductive health.

## METHOD

We conducted a literature search in PubMed, CINAHL, and EMBASE from 2007, when ENDS emerged in US markets, to October 2017, using the following search strings:

1) ‘pregnancy’ OR ‘pregnancy complications’ OR ‘pregnancy outcome’ OR ‘newborn’ OR ‘neonate’ OR ‘birth’; and 2) ‘electronic cigarettes’ OR ‘e-cigarettes’ OR ‘ecigarettes’ OR ‘vaping’ OR ‘vape’. We combined these using the Boolean AND operator. In November 2018 we repeated the search using Ovid MEDLINE for the period 2017–18. We also searched this database from 1946 to November 2018 for systematic reviews on smokeless tobacco and pregnancy outcomes. Repeated publications only were excluded. The authors independently reviewed the full text for inclusion and citations from the literature search.

## RESULTS

**Figure 1:**
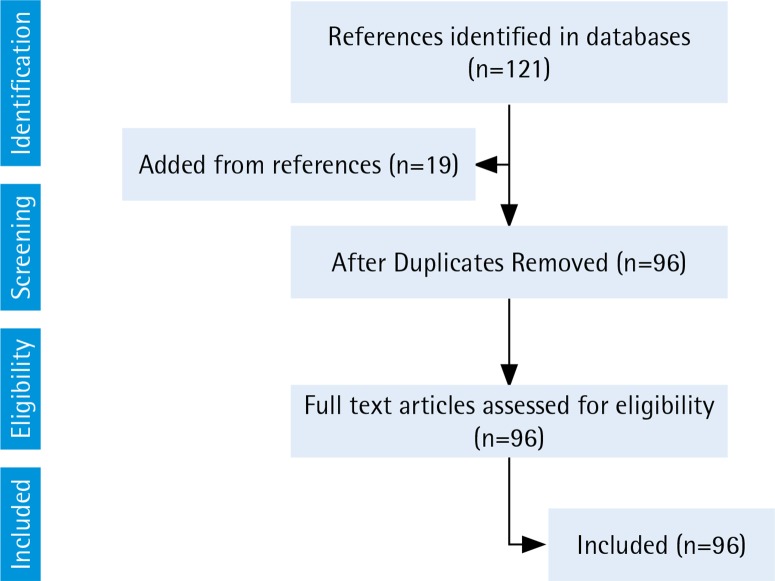
Manuscript selection: Electronic Nicotine Delivery Systems use in pregnancy

From 2007 to November 2018, a total of 96 distinct manuscripts were published pertaining to the topic of ENDS use and pregnancy or potential reproductive outcomes (Figure 1). We did not find any human studies that evaluated the effects of ENDS use during pregnancy on reproductive outcomes. One-third of the publications (n=34) consisted of reviews, with only 11 specifically addressing ENDS use during pregnancy or on the potential effects of ENDS use during pregnancy^8-19^. There were 21 reports of studies of fetal outcomes from pregnant animals or animal/tissue models, and 9 of these were designed to address ENDS prenatal exposure and its reproductive effects^20-26^. There was only one population-based study that estimated the prevalence of current ENDS use among pregnant women^27^. Other studies were conducted in prenatal clinics and failed to specify a case-definition of current use or excluded persons unfamiliar with ENDS^28-30^. We also found 10 reports on studies on determinants of ENDS use (including beliefs), trajectories of ENDS use during pregnancy^30-38^, and 3 publications on screening practices of practitioners^39-41^. We will examine each of these subtopics separately.

### Animal and bioassay studies

Experiments in pregnant rats that were given nicotine intraperitoneally showed that their pups had histone modifications that may maintain memory of nicotine exposure in the brain, while an epigenetic change in a splice variant of the glucocorticoid receptor of the lung was also observed^21^. In another study^24^, Balb/C pregnant mice were assigned to either sham, aerosols of ENDS with or without nicotine for 6 weeks before and during pregnancy and lactation. The offspring of ENDS with nicotine exposed pregnant mice showed short-term memory deficits, reduced anxiety, and hyperactivity, as well as global changes in DNA methylation, weight deficit, less fat, altered proinflammatory cytokines, and increased expression of neuropeptide Y and inducible isoform of NO synthase^20,24,25^. In a study^23^ on embryonic human stem cells and zebra fish, the effect of extracts of ENDS aerosols and cigarette smoke were examined. The authors found that both exposures caused a persistent delay in the differentiation of the mesoderm and reduced the expression of sarcomeric genes. An experiment on pregnant rats used a factorial design of *Mycoplasma pulmonis* and nicotine exposure. The data indicated that nicotine increased the risk of fetal infection, and resulted in a compromised placental barrier^22^. A study on embryos of the African clawed frog, *Xenopus laevis*, and a murine neural crest cell line, used a mixture of aerosols of ENDS that demonstrated the effect of ENDS use on defects such as median facial clefts and midface hypoplasia in embryos of *X. laevis*, and misexpression of cartilage and vascular genes in mammalian neural crest cells^26^.

### Prevalence and determinants of ENDS use during pregnancy

The 2013–2014 wave of the National Institute of Drug Addiction Population Assessment of Tobacco and Health study (the PATH study) included adult pregnant women. The PATH study, is a national probability household survey-based longitudinal cohort study of 45971 youth (aged 12–17 years) and adults in the US non-institutionalized population. Among the 388 pregnant women included in the study, 34 were current ENDS users, for a weighted ENDS use prevalence of 4.9%, (95% CI: 3.2–6.6). Current use was defined as ‘reported having ever used the product fairly regularly and using some days or every day now’. Twenty-eight of the current ENDS users (82.3%) were also current cigarette smokers (i.e. dual ENDS users)^27^. Subsequent waves of the PATH study provided estimates for the odds of quitting ENDS or cigarettes related to pregnancy that were higher for ENDS and hookahs than cigarette smoking^38^. In addition, the study has depicted the trajectories of use among ENDS dual users before pregnancy (Wave 1) and once they become pregnant (Wave 2); more than half (52.5%) stopped using ENDS once they became pregnant but continued to smoke cigarettes^37^.

Other studies reported the prevalence of ENDS use during pregnancy; the range of prevalence figures varied widely from the PATH study’s 5% estimate, possibly due to variations in how ENDS use during pregnancy was defined, how it was reported and the type of study population included. For instance, one study reported a 13% (42/316) prevalence of ever ENDS use among pregnant women seeking care at a university affiliated clinic in Maryland serving mostly African-Americans, but did not report the prevalence for current ENDS use^29^. Another study conducted at another US university affiliated prenatal care excluded pregnant women who were not aware of what ENDS use was and did not specify a definition for current ENDS use; in this study a prevalence of 12% was reported for current ENDS use during pregnancy^30^. A fourth study, using an online survey, estimated ENDS use in 15% of pregnant women. Current ENDS use in this study was undefined^28^.

Several studies, mostly in convenience samples of pregnant women offered counseling to stop smoking, have assessed beliefs and other potential risk factors for ENDS use. Among women of childbearing age in Central and Eastern Kentucky, who smoked cigarettes in the past year, ENDS use during pregnancy was considered less hazardous, use was more likely among younger and non-Hispanic Whites and driven by advertising^31,33^. In a qualitative study of Medicaid-eligible women seeking prenatal or post-partum care in Kentucky, participants believed that ENDS use decreased health risks, and those that reported dual ENDS use during pregnancy relapsed to cigarette smoking during the postpartum period^34^. The influence of advertisement on ENDS use was documented in an online survey that found that most (83.2%) women were aware of ENDS advertisements, and that viewing the advertisement increased their odds of considering ENDS to be safer than cigarette smoking in general (OR=2.5, 95% CI: 1.5–4.1) and for pregnant women (OR=2.1, 95% CI: 1.2–3.8)^28^.

A study of pregnant participants in a smoking cessation trial in Connecticut and Springfield Massachusetts reported a prevalence of ENDS current use of 14%. In this study, compared to never ENDS users, ENDS use was associated with a greater number of previous attempts to quit cigarette smoking, were more likely to self-identify as being Hispanic or non-Hispanic White, and having a drug addiction^32^.

The beliefs of pregnant ENDS users have been investigated extensively. One of the most informative studies consisted of 15 focus groups of women who were pregnant or planning to become pregnant, and were current smokers or had stopped smoking for the past 30 days. The focus groups were conducted at several locations in the US in 2013. Participants expressed their familiarity with ENDS and nicotine replacement therapy and showed more appeal to the use of ENDS than nicotine replacement therapy and specifically called ENDS ‘cute’ and ‘cool’, as portrayed by the tobacco industry^2^. Interestingly, the opinions regarding the safety of ENDS during pregnancy were divided, and some women expressed their reservations, while others considered them to be less harmful. Other studies have assessed the beliefs of pregnant women, and in general compared smokers to ENDS users. One systematic review, which focused on this subtopic, reviewed 7 studies that included a mix of pregnant and childbearing-age women. The review consistently found that women perceived that ENDS use during pregnancy carried less risk than cigarette smoking^16^. Since then, few other studies have replicated those findings^30^. A study using correspondence analysis found no correlation between the preferences for flavors and perceptions of harm for 50 pregnant ENDS users and 50 pregnant non-users living in southern New England^36^.

### Screening practices of providers

We found three surveys of screening practices by medical providers. In a mailed survey of US providers, 53% reported screening pregnant women at intake for use of ENDS and other emerging tobacco product use all or some of the time. Of these providers, 14% reported that ENDS use had no adverse health effects^39^. A second US survey, conducted in 2016 using the internet, targeted primary care practitioners at university affiliated practices, which reported that 61% asked pregnant women about their ENDS use^40^. In two surveys conducted in Australia, New Zealand, and the Torres Strait Islands, only 14% of general practitioners and obstetricians asked pregnant women most of the time about ENDS use^41^.

### Studies on the reproductive outcomes of smokeless tobacco

Since there are no previous human studies on the reproductive outcomes of ENDS use, and the effects of ENDS use alone is of interest for this review, systematic reviews on the reproductive effects of smokeless tobacco provide perspective and may be informative for hazard evaluation. According to the 2010 Global Adult Tobacco Survey conducted in 16 countries, smokeless tobacco use differs widely, being more prevalent among women than cigarette smoking^42^. In India and Bangladesh, where there is the heaviest burden of smokeless tobacco use, *Nicotina rustica* is primarily used instead of *Nicotiana tabacum* ^43^. Three systematic reviews focused on smokeless tobacco use during pregnancy, and its reproductive effects^44-46^. The first of these reviews focused on 21 publications and found evidence that smokeless tobacco use during pregnancy decreases the male to female live birth ratio, increases the risk of stillbirth, and results in low birth weight and maternal complications such as pre-eclampsia/eclampsia and anemia^44^. The second review included 9 studies and did not report summary estimates for the effects of smokeless tobacco on birth outcomes. This review concluded that there was substantial heterogeneity and that possible biases could explain these inconsistencies^45^. Finally, the third review was limited to two cohort studies conducted in populations in India, and focused on the effects of smokeless tobacco use during pregnancy on preterm birth, low birth weight and stillbirth. Results from these cohort studies indicated that there were increased odds of low birth weight, preterm birth and still birth among smokeless tobacco users^46^. In both of these cohort studies, the smokeless tobacco of interest was ‘mishri’, a powder prepared by roasting tobacco leaves^43^.

## DISCUSSION

As described in this literature review, the use of ENDS during pregnancy varies across studies. One population-based study estimated the prevalence to be approximately 5% in the US, which is consistent with the reported prevalence seen in US adults overall. However, higher prevalence figures (12–14%) have been observed in clinical populations and online. Also consistent with non-pregnant adult populations, most pregnant ENDS users as other adults are dual users, that is, concomitantly smoke cigarettes. However, dual use limits the ability of epidemiologic studies to estimate the independent effects of ENDS use on birth outcomes since the effects of smoking would be difficult to identify. Correspondingly, we did not find any published study that evaluated the effects of ENDS use on birth outcomes. However, studies on the reproductive effects of smokeless tobacco, which show harmful effects to the fetus, emphasize the importance of the need for future studies to be conducted to examine the effects of exposure to nicotine from ENDS use on the offspring of pregnant users.

Our systematic review of the literature also underscores the need for standardization of terms such as ‘current use’. For example, by only asking about current ENDS use during the past month, we cannot distinguish between temporary experimentation with ENDS and regular ENDS use^47^. It has been proposed to use frequency of use rather than use in the last 30 days to provide more informative data regarding the level of ENDS use^48,49^. The difference in the questions and the coding could explain the variations found in the prevalence of ENDS use among pregnant women (from 5% to 14%). In addition, self-reports of ENDS use could be also affected by an extension of the well-known non-disclosure of smoking use among pregnant women^50^, especially in non-clinical settings. Questionnaires need also to adapt to rapidly changing trends and new products such as the JUUL device. Studies that use biomarkers (possibly a combination of CO, cotinine and hair nicotine) in conjunction with questionnaire data to assess exposure to ENDS, instead of self-report alone, could minimize the impact of misclassification from non-disclosure^51^.

The evaluation of the potential impact of nicotine from ENDS use on human fetuses can also greatly benefit from the evaluation of intermediate outcomes such as DNA methylation, which was found altered in pregnant mice exposed to ENDS. Fetuses of smokers were found to have specific changes in DNA methylation possibly linked to intrauterine growth retardation and adult chronic disease^52-54^. The protective effect of folic acid supplementation in the prevention of neural tube defect is postulated to function through altered DNA methylation^55^.

## CONCLUSIONS

Since there are no current studies on the effects that ENDS use has on pregnancy outcomes, one can only hypothesize, based on existing studies on the reproductive effects of smokeless tobacco, that ENDS use by pregnant women is not safe for their fetuses. Given the need for studies of pregnant women who use ENDS, funding is urgently needed in support of studies on the health effects of ENDS use on birth outcomes. Pregnancy cohort studies are not only feasible, they also have a limited follow-up period, are less likely therefore to be affected by follow-up bias. Further, with relatively common outcomes such as smallness for gestational age and preterm delivery, only a relatively small study sample size is required, and could serve as the baseline for longer follow-up studies to assess child and adult health^56^.

## CONFLICTS OF INTEREST

The authors have completed and submitted the ICMJE Form for Disclosure of Potential Conflicts of Interest and none was reported.
